# The Repair of Wounds and Fractures in Aged Persons

**Published:** 1884-10

**Authors:** G. M. Humphrey


### The Repair of Wounds and Fractures in Aged Persons.

It is generally held that the healing processes are more protracted in the aged than in those of the early and middle periods of life. In the former the nutritive forces are failing, the strength and weight are diminishing, and repair is less and less able to keep pace with wear, and, therefore, it was a natural conclusion that the processes concerned in breach-closing should also show a decrease in energy. This opinion Professor Humphrey considers to be erroneous. He was led to investigate the subject by observing that ulcers in old persons frequently heal quickly, and that granulation and cicatrization proceed in them with great rapidity. Not wishing to rely entirely upon his own observations, he took pains to ascertain the opinion of most of the other surgeons of England, and found that they coincided exactly. Through the kindness of these gentlemen he was able to present a series of thirteen cases of wounds in elderly persons, their ages ranging from 75 to 93 years inclusive, where the healing was very rapid. He also reports a series of fifteen cases of fracture in old people, their ages being from 58 to 92 inclusive, where union was completed in a very short time. As an instance, we will cite the case of a woman, aged 81, who sustained a fracture of the thigh, below the middle, and also dislocation of the shoulder. In six week she could walk round the table with the help of a stick; in five months walked four miles.

In studying more closely this exceptional phenomenon of nutrition he found that not infrequently the very opposite tendencies existed at this time of life, namely, the tendency to slough and the tendency to heal quickly, and so the conclusion arrived at was, that when in elderly people the tendency to slough was absent, we could generally expect a rapid healing. This is not exceptional to old age, but may be observed in other lowered conditions. The wounds in patients exhausted by large losses of blood usually heal quickly, as they also do after operations for cancer, and in many other debilitated conditions, an exception must be made, however, of persons of naturally strumous temperament, and persons who have certain impaired conditions of the nervous system, in whom wounds and sores are sometimes very troublesome.—*G. M. Humphrey, F. R. S., British Medical Journal, July* 12, 1884.

